# Information needs and seeking behaviour among health professionals working at public hospital and health centres in Bahir Dar, Ethiopia

**DOI:** 10.1186/1472-6963-13-534

**Published:** 2013-12-27

**Authors:** Mulusew Andualem, Gashaw Kebede, Abera Kumie

**Affiliations:** 1 Department of Health Informatics, Institute of Public Health, University of Gondar, Gondar, Ethiopia; 2 School of Information Science, Addis Ababa University, Addis Ababa, Ethiopia; 3 School of Public health, Addis Ababa University, Addis Ababa, Ethiopia

**Keywords:** Information, Information need, Information seeking behaviour, Information source

## Abstract

**Background:**

Universal access to information for health professionals is a need to achieve “health for all strategy.” A large proportion of the population including health professionals have limited access to health information in resource limited countries. The aim of this study is to assess information needs among Ethiopian health professionals.

**Methods:**

A cross sectional quantitative study design complemented with qualitative method was conducted among 350 health care workers in Feburary26-June5/2012. Pretested self-administered questionnaire and observation checklist were used to collect data on different variables. Data entry and data analysis were done using Epi-Info version 3.5.1 and by SPSS version19, respectively. Descriptive statistics and multivariate regression analyses were applied to describe study objectives and identify the determinants of information seeking behaviours respectively. Odds ratio with 95% CI was used to assess the association between a factor and an outcome variable.

**Results:**

The majority of the respondents acknowledged the need of health information to their routine activities. About 54.0% of respondents lacked access to health information. Only 42.8% of respondents have access to internet sources. Important barriers to access information were geographical, organizational, personal, economic, educational status and time. About 58.0% of the respondents accessed information by referring their hard copies and asking senior staff. Age, sex, income, computer literacy and access, patient size, work experience and working site were significantly associated with information needs and seeking behaviour.

**Conclusions:**

The health information seeking behaviour of health professional was significant. The heaklth facilities had neither informationcenter such as library, nor internet facilities. Conducting training on managing health information, accessing computer and improving infrastructures are important interventions to facilitate evidence based descions.

## Background

Qualified health care professionals (HCPs) have vital roles in achieving health goals of a given country [1,2]. Updating knowledge with relevant information is very important for health care professionals to deliver quality and sustainable health care services to their consumers [1-3]. This is possible only when there is a sustainable access to health information resources (HIRs) in health facilities [4,5].

Health information is important to improve knowledge based on which evidence-based decision is made to serve the clients of health facilities. Access to health information facilitates to use new medical technologies, handle properly the necessary medical procedures and treatment of patients. The proper health information management brings health workers to act harmoniously in a similar manner on medical and health practice [3-5].

The relevance of health information is measured using two indicators. Information need: is a recognition that the knowledge is inadequate to satisfy a purpose that someone needs to achieve [6]. Information Seeking Behaviour: is the purposive searching for information because of a need to satisfy some goals.

Health professionals usually come accross with two basic HIRs: formal (hard and soft copies) and informal (human resources) [7-9]. Information needs and seeking behaviours varied among HPs working in rural and urban areas due limited accesse to information outlets [7,10]. Internet use, access to library, provision of training use of audios and videos displays were the means that provide health information to the users [4,11,12].

The use of health information may vary depending on circumstances. The need reaches pick during emergencies. Information needs of public health workforce had become more urgent and mandatory due to the emergency of new infectious diseases like severe acute respiratory syndrome (SARS), Asian bird flu, HIV/AIDS, malaria and tuberculosis. It was also due to the increasing concern of bioterrorism (spreading anthrax spores via the US Postal Service in 2001) [2,13,14].

Currently, resource limited countries face several health challenges that threaten the lives of millions of people [15]. Lack of health information communication creates such situations that produces medical errors, which are common in today’s health care organizations. This situation has the potential to cause miss-diagnosis, wrong treatment, increase multi drug resistance, severe injury and unexpected patients death [5].

Infrastructure, HIRs access, socio-demographic factors, cultural influences, poor initiation of users and geographical locations were some of the factors affecting the health information seeking behaviours of HPs in developing countries [16-18]. Based on research findings from Ethiopia [19], 146(94.6%) of one hundred sixty nine HPs sought information for their daily activities.

This study attempted to answer the following questions:

1. What HIRs were available in the study area?

2. What were the health information needs and seeking behaviours of HPs?

3. What is the coping mechanism when there is limited access to health information?

4. What were the factors affecting information seeking behaviour?

The findings of the study is expected to serve as a base line evidence for health administrators, policy makers, HPs, NGOs, and researchers to plan, take action and conduct intervention to improve HIRs access in the study area.

## Methods

Institutional based cross sectional quantitative study twith qualitative method was conducted in. The study was conducted in Bahir Dar town-a capital city of Amhara Regional State of Ethiopia. It is located in North West Ethiopia, about 565 Kms far away from Addis Ababa-a capital city of Ethiopia. Its climate is temperate and has many attractive touristic sites. The city has one public referral hospital, two private hospitals, four public health centres, more than 45 higher private clinics, more than 15 diagnostic laboratories and 30 drug distributing pharmacies.

Health workers employed in the public hospital and four Health Centres during the study period were source population for this study. Since the actual number of HPs in the study area was small, 350, all HPs were considered adequate for the study. The number of study subjects were 270 (77%) and 81 (23%), respectively, from hospital public hospital and 4 health centres. Pretested self-administered questionnaire was used to collect quantitative data. The questionnaire was developed by referring related studies (reference??). The tool contained questions related to socio-demographic characteristics, information seeking behaviour, HIRs access and information needs of HPs. The questionnaire was prepared in English, translated in to Amharic (local language) and then translated back to English to check the consistency in the language. 10% of the sample was pre-tested in a similar health facility that was outside the study area to check the validity.

Two data collectors and one supervisor participated in data collection. A one day training on the objective and relevance of the study, confidentiality of data, respondents’ right, informed consent, data collection techniques, and the content of the questionnaire was given prior to the data collection date. Consent was obtained for the administration of the respective health facilities.

Ethical clearance for this study was obtained from the Ethical Review Committee of the School of Public Health, Addis Ababa University. Letter of support was obtained from Amhara Regional Health Bureau and Bahir Dar town special Zone Health Bureau. Informed verbal consent was sought from individual study subjects for the willingness of providing data.

The self-administered questionnaire was distributed to study subjects through the facilitators of data collection with an assumption of getting back in a week time.

None participatory observation method using observation checklists (annex 1) was used to collect qualitative data by principal investigators. The observation technique was mainly focused on observing the presence of HIRs, information searching behaviour of HPs and the overall setups of the health facilities. The purpose of the qualitative data was to support the findings of quantitative method. Observation was done for consecutive three days to observe matters related to the organization of health information system.

Data collection methods were strictly followed to assure data quality. Data from the respondents were initially checked for completeness and consistency before data entry and cleaning using Epi Info Ver 3.5.1. Data was then exported to SPSS version 19 for analysis. Descriptive statistics was to describe the study population in relation to relevant variables. Binary logistic regression was computed to see the effect of each study variable on the outcome variable. Variables with significant effect were subjected in a multivariate logistic regression analysis to evaluate the consistency of the effect after adjusting other varaibles. The strength of associations was described using Odds ratio and 95% CI. Content analysis was used to analyse qualitative data according to the objective of the study.

## Results

### Socio-demographic characteristics of the study subjects

Of the total distributed questionnaires, 339 (96.9%) were completed, returned back and analysed. Non responses were attributed to lack of time and interest on the respondents side 222 (65%) of respondents were females. The mean (SD) age was 31 ± 5. About 45% of the respondents was in the age group of 26–30 years. Majority (76.1%) of the respondents were from the public Hospital (Table 1).

**Table 1:** Socio demographic characteristics of health workers in Bahir Dar, 2012

**Variables**	**Number (%)**
**Age:**	
21-25 years	32 (9.5%)
26-30 years	153 (45.1%)
31-35 years	96 (28.3%)
36-40 years	37 (10.9%)
41-45 years	19 (5.6%)
>46 + years	2 (0.6%)
**Sex:**	
Male	117 (34.5%)
Female	222 (65.5%)
**Professional category:**	
General practitioner	21 (6.2%)
Health officer	18 (5.3%)
Medical lab. personnel	38 (11.2%)
Nurse	151 (44.5%)
Pharmacy personnel	34 (10.0%)
Mid wives	27 (8.0%)
Others	50 (14.7%)
**Work experience:**	
≤6 years	185 (54.6%)
>6 years	154 (45.4%)
**Monthly income in Birr:**	
500-1000	4 (1.2%)
1001-1500	146 (43.1%)
1501-2000	45 (13.3%)
2001-2500	64 (18.9%)
2501-3000	37 (10.9%)
>3000	43 (12.7%)
**Family size:**	
≤4 members	258 (76.1%)
>4 members	81 (23.9%)

By profession, about 44.5% of HPs were nurses, followed by 38 (11.2%) laboratory and 34(10.0%) pharmacy personnel. One hundred forty six (43.1%) and sixty-four (18.9%) of the respondents had monthly income within the range of 1001–1500 and 2001–2500 Ethiopian Birr, respectively. More than half (54.6%) of the respondents had less than six years work experiences (Table 1).

Only 113 (33.3%) of HPs were computer literate and nearly one-third (36.6%) of them had access to computer at different areas; 26 (7.7%) at working area, 8 (2.4%) at home and 15 (4.4%) at both work area and home. About 29.8% of HPs had part time jobs outside their organization (Table 2).

**Table 2:** Computer access and literacy among HPs at study site, 2012

**Variable**	**Number (%)**
**Computer access:**	
Yes: at home	34 (10%)
at office	62 (18.3%)
at both	28 (8.3%)
No: Do not know	215 (63.4%)
**Computer use:**	
Report writing	1 (0.8%)
Reading	5 (4.0%)
File keeping	4 (3.2%)
Internet use	5 (4.0%)
Reading, file keeping, internet use	80 (64.5%)
All	29 (23.5%)
**Computer literacy(n = 339):**	
Yes	113 (33.3%)
No	226 (66.7%)
**Computer illiteracy reasons(n = 226):**	
No time	51 (22.6%)
No access	135(59.7%)
Not interested	2(0.9%)
No time & no access	38 (16.8%)
**Do you have other means of income (n = 339)**	
Yes	101(29.8%)
No	238(70.2%
**Time spent after regular work(n = 228):**	
With my boy/girl friend	65 (27.7%)
Visiting my socials	42 (17.7%)
Through reading	85 (35.7%)
Church	16 (6.7%)
Recreational place	15 (6.3%)
Others	15 (6.3%)

### Information needs of health professionals

Majority (97.3%) of HPs needed information to update knowledge to support their daily activities. Formal HIRs were preferred by 98.8% of HPs than informal HIRs (Table 3). Self-initiated information needs and question from patients (56%) were the major reasons for HPs to seek HIRs. Only 145 (42.8%) of the respondents had access to internet/searching on Google engine/at different places with variable frequencies (Table 4).

**Table 3:** Purpose/reasons of using HIRs among health professionals in Bahir Dar, 2012

**Variable**	**Number (%)**
**Health information needs:**	
Yes	333 (97.3%)
No	9 (2.7%)
**Reasons for health information needs:**	
Own needs	87 (25.7%)
Questions from patients	21 (6.2%)
Environmental competitions	5 (1.4%)
Emergency of new cases	6 (1.7%)
Own needs, questions from patients & new cases	31 (9.1%)
Own needs & questions from patients	190 (56.0%)
**Problems encountered due to information limitation:**	
Yes	183 (54.0%)
No	156 (46.0%)
**Purposes to use internet:**	
E-mail	90 (62.0%)
Patient care & drug information	37 (25.5%)
New findings	13 (9.0%)
Research & business	5 (3.5%)
**Satisfaction on internet:**	
Partially satisfied	18 (12.4%)
Not satisfied	127 (87.6%)
**Problems encountered while searching:**	
Poor internet connection	80 (55.2%)
High cost	45 (31.0%)
Poor searching skills	20 (13.8%)
**Training time:**	
Within the last 6 months	78 (37.0%)
Within the last3 months	70 (33.2%)
Within the last 12 months	26 (12.3%)
More than 12 months	24 (11.4%)
Do not remember the time	13 (6.1%)
**Need for further training:**	
Yes	325 (96.0%)
No	14 (4.0%)

**Table 4:** Health Information Resources access among HPs in Bahir Dar, 2012

**Variables**	**Number (%)**
**HIRs accessed (n = 339):**	
Formal HIRs (books, internet, trainings, etc.)	315 (92.9%)
Informal HIRs (key informants, colleagues, etc.)	24 (7.1%)
**Library access at working area:**	
Yes	252 (74.3%)
No	87 (25.7%)
**Books, Journals, etc. accessibility at working place:**	
Yes	31 (9.1%)
No	308 (90.9%)
**Information sources preferred (n = 339):**	
Formal HIRs	335 (98.8%)
Informal HIRs	4 (1.2%)
**HIRs at home :**	
Yes	275 (81.1%)
No	64 (18.9%)
**Are HIRs at home use full?**	
Yes	214 (77.8%)
No	61 (22.2%)
**Training access:**	
Yes	211 (62.2%)
No	128 (37.8%)
**Internet access:**	
Yes	145 (42.8%)
No	194 (57.2%)
**Internet sources:**	
Working area	46 (31.7%)
Internet café	55 (38.0%)
Home	21 (14.5%)
Either from two or three of above sources	23 (15.8&)
**Mechanisms of solving information related problems:**	
Consulting seniors	57 (31.1%)
Discussion with staffs	58 (31.7%)
Referring patients	6 (3.3%)
Consulting & discussion with staffs	34 (18.6%)
Discussion with staffs & refereeing patients	1 (0.6%)
Consulting & discussion with staffs & refereeing patients	22 (12.0%)
All	5 (2.7%)

### Health information resources access and seeking behaviour

**Figure 1:**
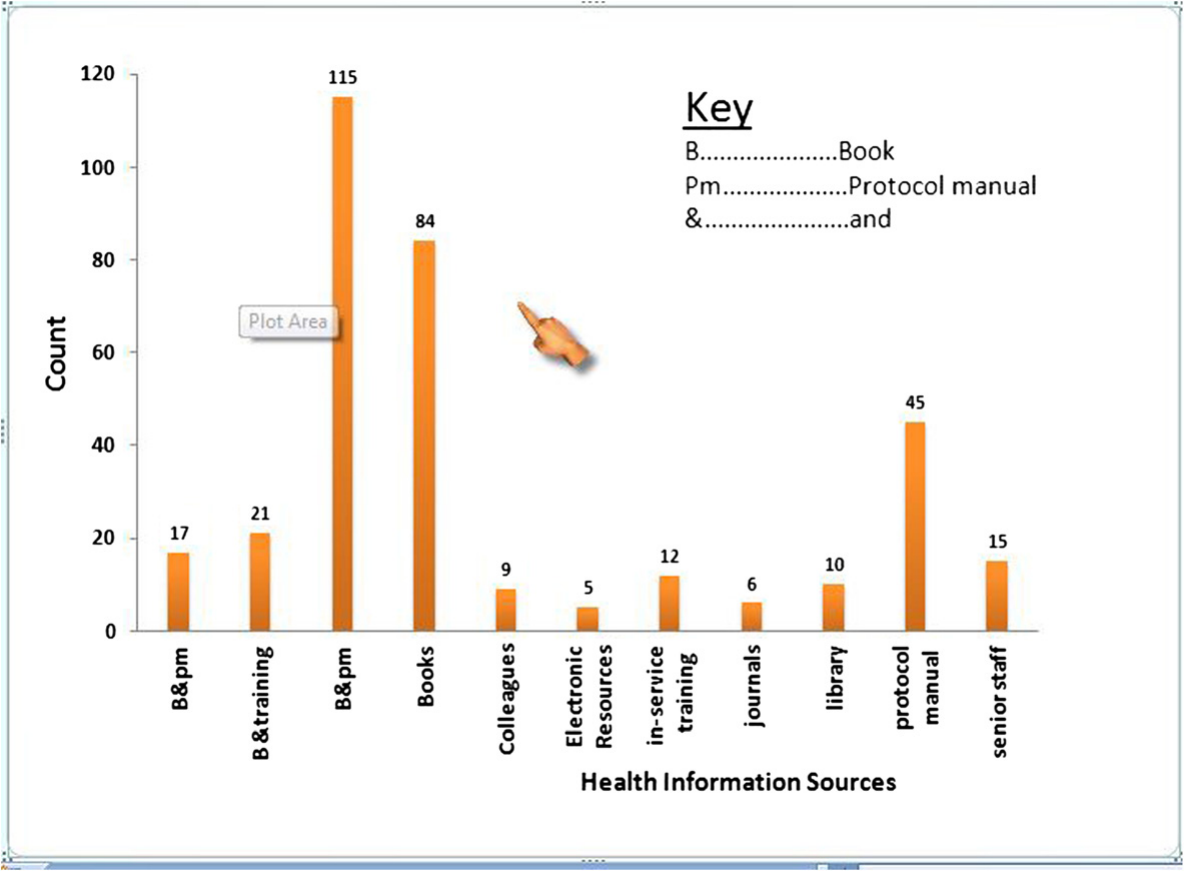
Information resources accessed by HPs in Bahir Dar, 2012.

TB-HIV, Malaria, patient diagnosis, PMTCT, under five, ART, quality lab results, drug administration and new findings were mentioned areas for seeking HIRs. Accessed HIRs by HPs were text books and protocol manuals 115 (39.3%), only books 84 (24.8%) and only protocol manuals 45 (13.3%) (Figure 1). Accessed internet included various sources. Major sources of internet services for HPs were working area 46 (31.7%), internet cafe 55 (38.0%) and at home 21 (14.5%) (Table 4). Common reasons for using internet among participants were e-mail 90 (62.0%), drug and patient care information 37 (25.5%) and new findings 13 (9.0%).The most frequently used internet search engine by HPs was Google engine (Table 3).

Even though 74.3% HPs from referral hospital had access to mini-library within their working environment, only seven (5.0%) of the respondents were satisfied with the services given from mini library. More than half (56.9%) of respondents did not do anything to make HIRs available in their working areas. Thirty-one HPs reported that their organization had subscribed HIRs like books, journals and standard manuals. Concerning usefulness of those subscribed resources, only three participants reported that they were fully useful. About 276 (81.1%) study participants had HIRs at their home. Majority (77.8%) of the respondents who had owned HIRs at home reported that their HIRs were recent and relevant for using (Table 4).

Majority (87.6%) of the respondents were not satisfied with the internet they had accessed. Of those who had access to internet, 122 (84.1%) encountered problems like poor internet connection 80 (55.2%), high cost 45 (31.0%) and poor searching skills 20 (13.8%). More than half (62.2%) of HPs had taken in service trainings on information management in the past (Table 4). About 183 (54.0%) HPs had encountered information gaps on their work due to information limitation (Table 3). They were using different mechanisms to solve the gap such as conducting a discussion among their staff (31.7%) and consulting their senior staffs (31.1%) (Table 4).

Association between internet access/major HIRs/and majority of other independent variables was observed (Table 5). In the crude analysis, respondents with age group < 30 years of age had higher likely hood of accessing internet than to those workers who have age ≥30 years, [OR = 3.7, 95% CI (1.81, 7.63)].

**Table 5:** Association of internet access with study variables, 2012

**Internet access**					
**Variables**		**Yes (%)**	**No (%)**	**COR (95%CI)**	**AOR(95%CI)**
**Age:**	<30 years	100 (29.5%)	85 (25.1%)	3.7[1.81,7.63]^*^	2,63[1.4,5.1]
	≥30 years	14 (4.1%)	44 (13.0%)	1.0	1.0
**Sex:**					
Male		63 (18.6%)	54 (15.9%)	2[1.3,3.14]^*^	1.91[1.1,3.4]
Female		82 (24.2%)	140 (41.3%)	1.0	1.0
**Educational category:**					
MD		20 (5.9%)	1(0.3%)	2[1.3,3.14]	1.91[1.1,3.4]
Others		125 (36.9%)	193 (56.9%)	1.0	1.0
**Working unit:**					
OPD & inpatient		32 (9.4%)	33 (9.7%)	1.38[0.80,2.4	1.31[0.64,2.66]
Others		113 (33.3%)	161 (47.5%)	1.0	1.0
**Monthly income:**					
<2000		58 (17.1%)	137 (40.4%)	0.3[0.18,0.44]	0.38[0.22,0.68]^*^
≥2000		87 (25.7%)	57 (16.8%)	3[0.18,0.44]^*^	2.0[1.1,3.87]
**Working experience:**					
≤6 years		101(29.8%)	84(24.8%)	3[1.9,4.86]^*^	2.0[1.1,3.71]
>6 years		44(13.0%)	110(32.4%)	1.0	1.0
Family size	<4	117(34.5%)	141(41.6%)	1.6[0.91,2.73]	0.77[0.39,1.5]
	≥4	28(8.3%)	53(15.6%)	1.0	1.0
**Computer access:**					
Yes		80(23.6%)	42(24.4%)	2[1.3,3.30]	1.8[1.03,3.10]
No		106(31.3%)	103(30.4%)	1.0	1.0
**Patient number per day:**					
≤30		88(23.6%)	44(13.0%)	2[1.3,3.30]	1.8[1.03,3.10]
>30		65(19.2%)	150(44.2%)	1.0	1.0
**Computer literacy:**					
Yes		72(21.2%)	41(12.1%)	3.7[2.23,6.1]	3.93[2.26,6.82]^*^
No		73(21.5%)	153(45.1%)	1.0	1.0
**Facility:**					
Hospital		124(36.6%)	134(39.5%)	2.64[1.5,4.8]^*^	2,59[1.33,5.01]
Health centers		21(6.2%)	60(17.7%)	1.0	1.0

^*^Significant at p < 0.05.

### Factors affecting health information seeking behaviour

The most frequently mentioned hindering factors from both approaches were geographical, economical, organizational, educational status, poor personal initiation, governmental/policy related, low prevalence of new cases and to some extent time shortage.

### Qualitative analysis results

#### Health information resources access and utilization at feleg hiwot referral hospital

There was only one mini library (approximately 4×4 meter area) inside the hospital. There were few too old books, few copies of certain text books, too few old journals and eight (6 functional and 2 non-functional) desktop computers with wireless internet connection within that library. There were manuals and standard guidelines in few wards. ART laboratory, VCT, ART, and TB-HIV clinics were better in access of standard guidelines, charts and reporting formats. Some banners and case definitions for few cases such as measles, polio, acute diarrhoea, TB, STIs and malaria were posted on some ward walls. There were few desktops only in mini library, Laboratory, pharmacy, ART clinic, TB clinic and VCT rooms. The hospital has one-morning session/cases presentation program per week for HPs. Consulting of specialists and senior staffs, five-four personal computers and few soft copy materials among General Practitioners (GPs) were observed during data collection period.

Majority of HPs from hospital were not using HIRs frequently to to improve knowledge. Only few (4–5) HPs were using standard guidelines, especially national treatment guidelines to order treatment for their patients. Very few HPs who have class to update their level were using their handouts during observation period.

Most of HPs were observed spending extra time simply by side talking in groups and moving around. On average, six- to eight HPs were observed while using internet and books in the library. Two-third of them were using internet for e-mail and chatting purpose. Most HPs had mobile phones with internet and were using it for face book chatting. From those who used internet, more than 90% were young GPs with the approximate age group of 27–30 years. Library personnel determined the average stay time for one person at one computer for only 30 minutes per session in order to save time for others.

#### Health information resources access and utilization at health centres

Almost all health centres have similar infrastructure and services in terms of handling their HPs. There was no library, internet, journals, books, computer and computer rooms, research papers, soft copy materials, peer/group discussion, training access during observation period in all health centres. Few handouts, training manuals and few standard guidelines were observed among few HPs.

There were consultation practices and one Ethiopian TV service almost in all health centres. One morning session among HPs was observed only at Abay health centre. Three-four HPs/degree candidates/were using their handouts, text books and exam sheets in each health centres during observation period. Two-three HPs working in ART and TB-HIV clinics were discussing each other in Bahir Dar and Han health centres.

## Discussion

This study indicated that almost all study participants (97.3%) had information needs This finding was consistent with study findings from Addis Ababa [18] where the overall information needs of HPs was 94.8%. Various studies conducted on HI needs and seeking behaviours showed that the majority HPs needed to access HIRs for the provision of quality health care services to their clients [11,16,17,19,20].

However, poor personal initiation, poor HI seeking and utilization of available information was observed in this study. More than half of the respondents (56.9%) did not inquired library services at their working areas. This is supported by related study conducted in one of the district in India [21] where awareness creation on HIRs, how to access and utilise them were recommended. Unavailability of HIRs was mentioned to be the most important reason for the presence of poor HI seeking behaviours among HPs in this study. This in line with study findings from Addis Ababa [19,22].

Protocol manuals, health text books, consultation and in-service trainings were reported to be the most frequently used HIRs; 115 (39.3%) followed by books 84(24.8%). Electronic sources were the least used HI sources (1.5%) in the study area. These findings were almost similar with study findings from Addis Ababa [19]. These findings were different from study findings in Nigeria [16] where the frequently used HIRs were medical textbooks, journals, discussion with colleagues and internet searching. It was also different from study findings in Uganda where frequently accessed HIRs among HPs were discussions with colleagues (89%), medical textbooks (77%) and (29%) both internet and libraries [11,22,23].

In our study, discussion with colleagues was poorly practiced (2.7%) which was in contrast to findings from Uganda (89.0%). The possible reasons might be due to poor reading culture, giving less attention to it, lack of openness among staff, fear of criticism, giving priorities for other issues than profession a concerns.

Using electronic resources, research, report papers and journals as HI sources among HPs in the study area was too poor in this study which was similar with findings from China, Egypt, Kenya, India, and Thailand hospital doctors where hard copies and textbooks were the most commonly used HIRs [24]. However, the findings in this regards was different from study findings in Addis Ababa [25], where major preferred HIRs among GPs were printed journals (29%) and electronic resources, CD-ROMS (22%) [25]. The probable reasons for the above may be absence of library access, poor initiations of HPs, no research activities among staffs (100%), no feedback/copies of research papers from different investigators who conducted research activities at those organizations (100%), no computer and internet access at all health centres (100%). These reasons were consistent with the study conducted in Addis Ababa [19] and Iran [23] where scarcity of budget, time shortage, computer and room shortage and lack of skills were identified barriers.

Currently, ICTs (E-mail, mobile phones and Internet) are playing vital roles in effective information dissemination among HPs located in different parts of the world within fraction of stime and minimum cost [4,5,14,15]. In the current study, only 113 (33.3%) of total HPs were computer literate. This figure was much lower as compared with study findings from Ibadan, Nigeria [26] where 93% of physicians were computer literate. It was also slightly lower compared with the study findings from Addis Ababa hospitals and health centres [19,25] where 46.7% of respondents were computer literate. The major identified reasons for the presence of high computer illiteracy rate (66.7%) in the study area include limited access to computer system 136 (60.2%) and time shortage 51(22.6%). These figures were larger than study findings from Nigeria [26]; only 7% computer-illiterates with similar reasons as our study. It was also quite different from study findings in Addis Ababa [19], where computer illiteracy rate was 53.3% with the same reasons.

In this study, only 145 (42.8%) study participants had internet access at one or more areas, which is lower by half compared with study findings in UK [27]. It was also lower than study findings from Addis Ababa [25] where major internet sources were internet café (42%), at working areas (34%) and at home (23%). Low access to internet in the study was mainly due to the absence of internet services in all health centres, poor connection in hospital, too few computer accesses in each study site, low computer literacy rate, poor personal initiation and high cost of internet connection. Most of these reasons were supported by study findings from hospitals and health centres in Addis Ababa [19,25].

Majority (57.7%) of respondents got information by consulting their text books, medical protocol manuals, training handouts, discussing with senior staffs. This finding was supported by different studies where most of them had a habit of using printed resources as first choice HI source [11,16,17,19,20].

Top major identified cahllenges to access HIRs in the study area were geographical, organizational, economic related, educational status, poor personal initiation, time shortage, low prevalence of new cases. This was in line with study findings by Anwar F & Shamim A [28] on assessing hindering factors of HI Technology (HIT) for developing countries where major barriers were infrastructure, cost and time, lack of national policies towards HIT, social and cultural barriers, educational and organizational barriers [17-19,24,29].

It was found that more than half (54%) of HPs were encountered with problems during their daily activities due to information limitation. This large figure showed that there were larger information gaps among HPs. It was supported by different studies done in related topics among health care service delivery facilities in developing countries [2,5,15,16,19].

## Conclusions

In conclusion, almost all HPs felt to have limited access to HIRs. Most HPs have limited or no access to major HIRs, especially on formal information resources likes internet, journals, library service, and in-service training. Majority of HPs were doing their activities based on their school knowledge and some on job trainings. The most commonly accessed HIRs in all health facilities were consultation of senior staffs and on job trainings. The most important identified factors for the presence of poor HIRs access in the study area were poor personal initiation, organizational, economic, poor information culture, skill related, educational status and to some extent time shortage. Conducting training on HIRs, improving infrastructures, accessing computer and internet services and peer education are important to solve the problem. Exploring further the effective ways of increasing HIRs access in the study area is a research agenda in countries with limited resources.

## Competing interests

The authors declare that they have no competing interests.

## Authors’ contributions

MA (PI) participated in all steps of the study from its commencement to paper write up and manuscript preparation. AK and GK participated in a proposal development, data management and analysis, manuscript preparation and editing. We authors have reviewed and approved the submission of the manuscript. All authors read and approved the final manuscript.

## Pre-publication history

The pre-publication history for this paper can be accessed here:

http://www.biomedcentral.com/1472-6963/13/534/prepub
